# A Text-Book of Obstetrics

**Published:** 1904-09

**Authors:** 


**A Text-book of Obstetrics.** Edited by Richard C. Norris, M.D. Robert L. Dickinson, M.D., Art Editor. Second Edition. Two vols. Pp. 554, 547. London: W. B. Saunders & Co. 1902.

This work is a text-book written jointly by several of the best-known American obstetricians, and forms a valuable addition to the literature of the subject.

It is concisely written, but at the same time treats with considerable detail the more important portions of the subject. The student will find in the theoretical portion a large amount of valuable information based on recent researches, while the practitioner will find in the more practical portion valuable hints and explanations of the treatment recommended.

The illustrations, many of which are taken from photographs, are numerous and clear, and several not previously embodied in text-books on this subject will well repay careful study.

The section on the development of the ovum is especially noteworthy in this respect, the illustrations of the early development of the fœtus being most complete, and serving to make clear some of the more difficult points connected with early embryology.

We observe that the author of this section has adopted the views of Peters as to the attachment of the ovum, namely that it practically becomes buried in the maternal decidua by a burrowing process, in the course of which a portion of the maternal decidua is excavated and probably absorbed by the ovum. The structure and development of the placenta are described with great detail and accuracy.

We notice in the practical part the importance which the author appears to attach to the operation of episiotomy. In this, we think, many British authorities differ from him, both as to its efficacy in preventing lacerations and also as to its necessity. Certainly the wound so produced is not a difficult one to close by suture afterwards; to us its only advantage appears that it is rather more easily accessible than the ordinary laceration.

The remark that "operative midwifery is a department of surgery governed by the principles and rules of surgery" is one that, in our opinion, cannot be too strongly emphasised. At the same time, in laying down the rules for practitioners, especially with reference to those dealing with aseptic and antiseptic technique, we note with approval that while the importance of a safe minimum, and that one within the reach of any practitioner, is insisted on, too great complication, and the aiming at theoretical perfection in private practice, is deprecated: for this reason pure aseptic technique is rejected except in hospital practice, and reliance placed on the proper use of antiseptics.

The startling statement, which we are aware is to some extent borne out by certain British records, is made, that of women confined in private practice one in from 89 to 100 dies of septic infection, while the mortality in hospital is less than a fifth of this.

It is curious to note that in one chapter "auto-infection" is spoken of as a counsel of despair, and its occurrence doubted; while in another it is mentioned as being due to certain precedent maternal conditions. The explanation of this apparent contradiction is that the two sections are by different authors; this, however, is qualified in regard to the first of these statements by the addition "auto-infection in the common acceptance of the term," in which form the apparently contradictory statements are reconciled.

We can thoroughly recommend the work as reliable and interesting.

